# Development and pharmaceutical investigation of novel cervical cancer-targeting and redox-responsive melittin conjugates

**DOI:** 10.1038/s41598-023-45537-x

**Published:** 2023-10-25

**Authors:** Seray Sahsuvar, Rabia Guner, Ozgul Gok, Ozge Can

**Affiliations:** 1 Department of Medical Biotechnology, Institute of Health Sciences, Acibadem Mehmet Ali Aydinlar University, Istanbul, Turkey; 2 Department of Biomedical Engineering, Graduate School of Natural and Applied Sciences, Acibadem Mehmet Ali Aydinlar University, Istanbul, Turkey; 3 Department of Biomedical Engineering, Faculty of Engineering and Natural Sciences, Acibadem Mehmet Ali Aydinlar University, Icerenkoy, Kayisdagi Cd., Atasehir, 34752 Istanbul, Turkey

**Keywords:** Biotechnology, Cancer, Drug discovery

## Abstract

Cervical cancer has recently become one of the most prevalent cancers among women throughout the world. Traditional cancer therapies generate side effects due to off-target toxicity. Thus, novel cancer medications coupled with suitable drug delivery systems are required to improve cancer therapies. Melittin peptide has a high affinity to disrupt cancer cells. In this study, we designed targeted and redox-responsive Melittin conjugates for cervical cancer and then tested them in vitro. Folic acid and squamous cell carcinoma-specific peptide (CKQNLAEG) were used as targeting agents to design various conjugates. Our findings indicate that both anticancer conjugates were effective against different cancer cell lines, including MCF-7, C33A, and HeLa. Moreover, these conjugates were found to have antioxidant and antibacterial effects as well as reduced hemolytic activity. The CM–Target (N-terminus cysteine modified-Melittin–targeting peptide-functionalized conjugate) has become more stable and acted specifically against squamous cell carcinoma, whereas folic acid (FA)–containing conjugates acted efficiently against all cancer types studied, especially for breast cancer. According to our results, these anticancer conjugates may be possible anticancer drug candidates that have fewer adverse effects.

## Introduction

Recent epidemiological reports show that cervical cancer is one of the most common cancer types among women worldwide^1^. Squamous cell carcinoma and adenocarcinoma are the most common cancer subtypes, and account for 70% and 25% of all cervical malignancies, respectively. High-risk subtypes (i.e., 16–18 types) of the human papillomavirus (HPV) constitute the root cause of this disease in most cases^2^. Cancer therapies such as chemotherapy and radiotherapy, which are used in many conventional cancer treatments, can lead to common adverse effects due to off-target cytotoxicity and can damage other healthy tissues^3^. Therefore, next-generation cancer drugs combined with more specific drug delivery systems are needed to improve cancer therapies.

The use of peptide-based medicines inspired by nature has become more common in cancer therapies. Honeybees (*Apis mellifera*) produce a complex venom, the majority of which consists of peptides. Melittin, one of the main components in bee venom, is a α-helix peptide containing 26 amino acids (GIGAVLKVLTTGLPALISWIKRKRQQ)^4^. Under normal physiological conditions, Melittin causes changes in the structural integrity of the membrane when bound to the membrane surfaces of the cell. Melittin binds exclusively to phosphatidylcholine membranes^5^ but has an even higher affinity to negatively-charged membranes in cancer microenvironments. Thus, Melittin has a stronger affinity for the membranes of cancer cells, where anionic phospholipids are abundant, relative to healthy cells^6^.

Understanding the cancer microenvironment is crucial for the development of targeting conjugates. Some of the changes in this microenvironment include low pH^7^, high temperature^8^, and overexpressed glutathione (GSH)^9^. Generally, the cytosolic GSH levels in cancer cells are at least four times higher than in normal cells^10^. This difference in GSH levels between cancer and healthy cells make it possible to design redox-responsive conjugates. This feature depends on the level of GSH, especially in conjugates containing disulfide bonds. When the GSH level increases, the disulfide bonds can be broken, thereby allowing the drug molecule to enter the cancer cell as cargo. In addition, since the extracellular environment has 1000-fold less GSH level than the intracellular environment, this difference in GSH levels breaks the disulfide bonds and induces the release of the cargo molecule into the cancer cell in a safe way^11^. Previous research has shown that polymer/Melittin conjugates containing disulfide bonds can be transported to cancer cells effectively and can release the Melittin peptide to which it is bound due to high intracellular redox levels^12^. In addition to disulfide bonds, it has been observed that peptides containing thiol in the terminal group can cross-react with cell surface thiols^13^. Therefore, it would be expected that after the disulfide bonds in our conjugates are broken, the liberated Melittin peptide will be uptaken by cancer cells via efficient endocytosis.

To design cancer-targeted agents, we focused on receptors that are expressed at higher levels in cancer cells than in healthy cells. The folate receptor (FR) is a well-known biomarker due to its overexpression in many cancer subtypes such as cervical cancer. The binding of folic acid (FA) to FR with high affinity makes it a suitable targeting agent for cancer drugs^14^. On the other hand, a second targeting agent in peptide form was determined to compare the effectiveness of targeting molecules. The researchers identified the CSP-KQ peptide (CKQNLAEG), which is specific to cervical squamous cell carcinoma tissue, as a result of screenings on human cervical cancer xenografts with phage display peptide libraries. The in vivo tumor-targeting potency of the peptide was then determined by injecting it into mice via cervical cancer xenograft^15^. In our study, FA and CSP-KQ peptide were selected as targeting groups and coupled to Melittin via a redox-sensitive linker containing a polyethylene glycol (PEG) polymer. To determine the effects of these novel conjugates on cancer cells, cell lines were selected based on their purpose.

HeLa cells are HPV-induced cervical adenocarcinoma, whereas C33A cells are HPV-negative cervical squamous carcinoma^16^. The reason for choosing these cell lines was to control the specificity of CSP-KQ targeting peptide and to understand the general effect of FA–containing conjugates on different cell types. In addition to cervical cancer cells, we wanted to test our conjugates in other cancer type. Our expectation for targeting peptide–containing conjugates was less affinity for MCF-7 cells. Conversely, our expectation is that the activity of FA-containing conjugates remains the same or increases, since MCF-7 is the FR positive cell line^17^. 3T3 and NSF cells are healthy mouse and healthy human fibroblast cells, respectively. These cell lines are often used as a control group to see the effect of cancer drugs on healthy cells^18,19^. Accordingly, 3T3 cells were used to lead in vivo experiments, while NSF cells were used for in vitro experiments.

The aim of this study was to develop conjugates of Melittin peptide, which has high anticancer potential, that would reduce its side effects without much lowering its anticancer activity and make it more stable and targeted. For this purpose, targeting molecules were conjugated from either the N- or C-terminus since it could not be predicted which end of Melittin was more active. Following the analysis of the produced conjugates, in vitro tests were performed to assess their pharmacological effects. While cytotoxicity and reactive oxygen species (ROS) assays were investigated the anticancer and antioxidant effects of conjugates on cancer cells, several experiments were also conducted to understand their antibacterial and hemolytic/plasma protease activities.

## Results

For all in vitro experiments, calculations of tested drug concentrations were made according to the modified Melittin (Mel) peptide concentration present in the conjugate. N-terminus cysteine modified-Melittin–folic acid functionalized conjugate (CM–FA), C-terminus cysteine modified-Melittin–folic acid functionalized conjugate (MC–FA), N-terminus cysteine modified-Melittin–targeting peptide-functionalized conjugate (CM–Target), and C-terminus cysteine modified-Melittin–targeting peptide-functionalized conjugate (MC–Target) were tested in vitro.

**Figure 1:**
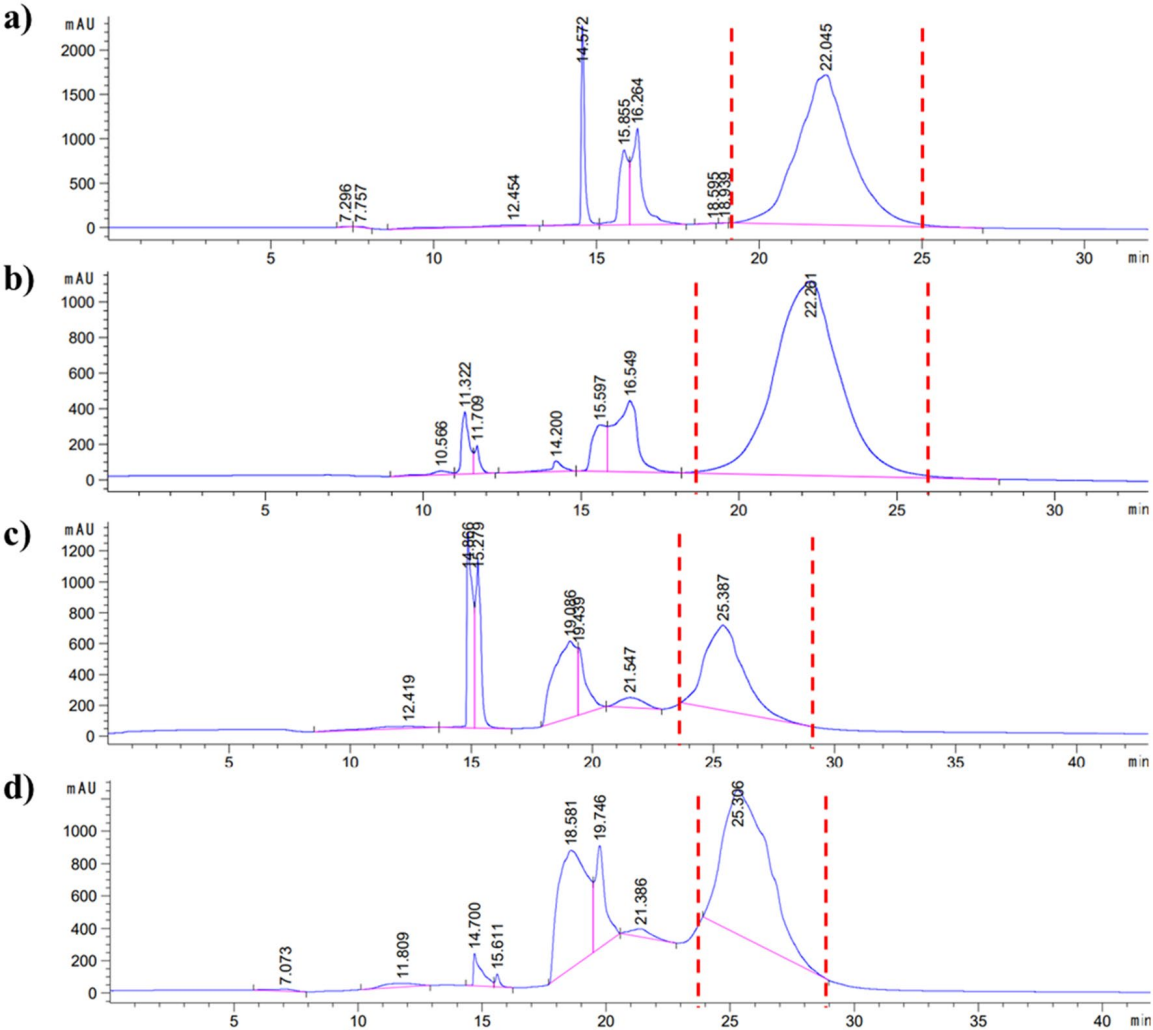
Semi-preparative HPLC chromatogram of the conjugates during purification shown as (**a**) CM–FA, (**b**) MC–FA, (**c**) CM–Target, and (**d**) MC–Target.

**Figure 2:**
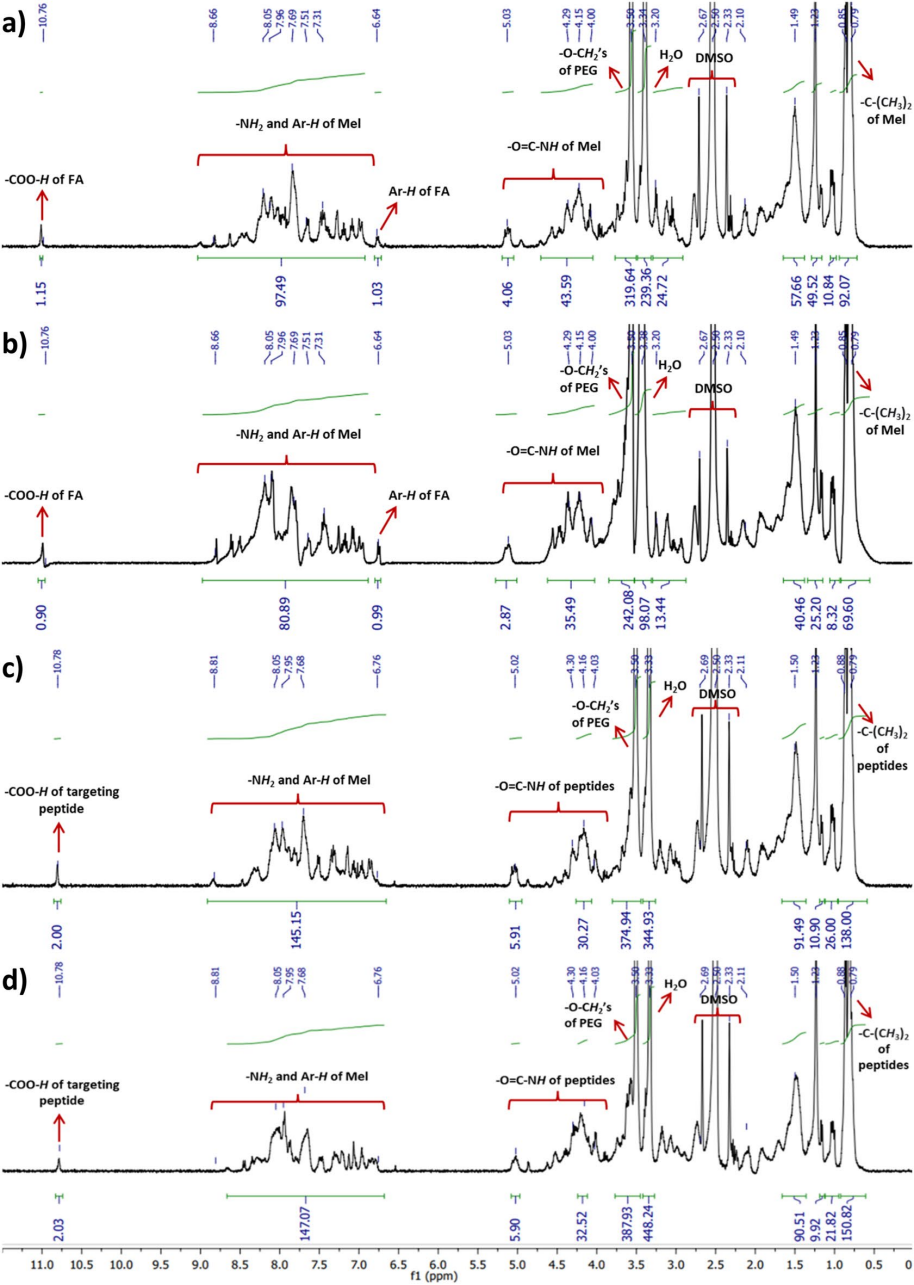
Proton NMR spectra of the synthesized conjugates shown as (**a**) CM–FA, (**b**) MC–FA, (**c**) CM–Target, and (**d**) MC–Target.

**Figure 3:**
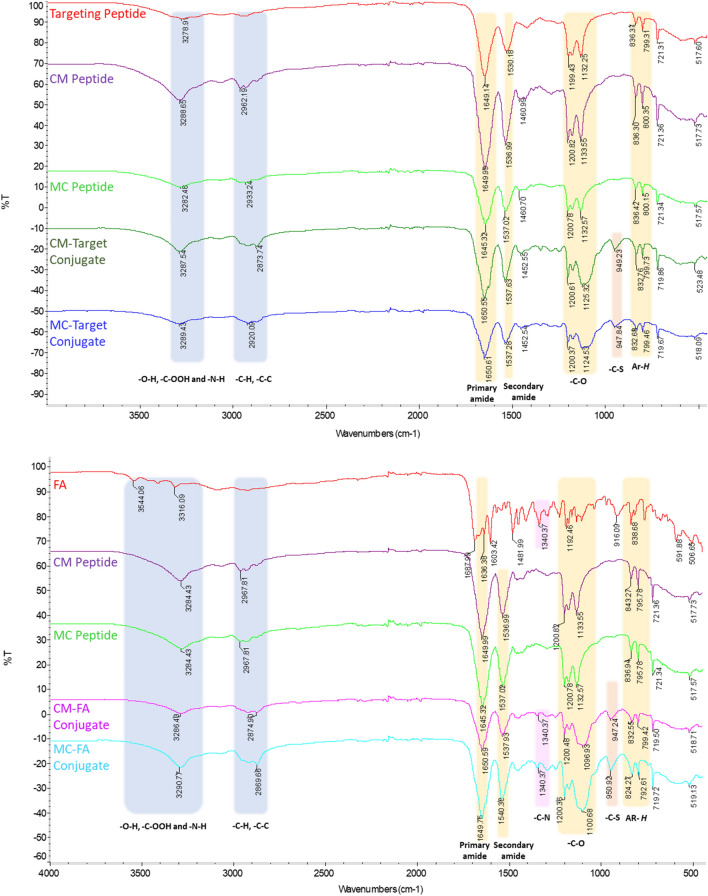
FT-IR spectra of FA, the peptides, and conjugates. The wavenumbers shown between 4000–500 cm^−1^, and all bonds indicated in the spectra.

The peptides and conjugates were purified using HPLC and all peaks from HPLC chromatograms were collected (Fig. 1, Supp. Data Fig. 1, Supp. Data Table 1). Then, FT-IR and NMR analyses were performed for the collected peaks. These analyses revealed that the red dashed peaks indicated the desired conjugate forms. In FA–containing conjugates, the red dashed peaks corresponded at 60–80% ACN while targeting peptide-containing conjugates were at 58–70% ACN (Fig. 1). When the naked Mel peptide was functionalized with a cysteine group at its N- or C-terminus, its hydrophobicity increased from 67% ACN to 78% ACN. Moreover, the targeting peptide was found to be more hydrophilic than other peptides, as shown in Supp. Data Fig. 1. In addition, the targeting peptide-containing conjugates were more hydrophobic than the FA–containing conjugates. Also, since all conjugates have linkers containing PEG(2K), a hydrophilic polymer, their hydrophobicity is reduced compared to the naked peptide. In addition, the purity of the collected peptide/conjugate peaks was analyzed by loading the column again (peaks purity: > 95, data not shown). Peptides were analyzed before conjugation reactions using LC–MS/MS. Peptide fragments were determined after ionization by their mass/charge (m/z) ratio from the + ESI scan spectrum (Supp. Data, Fig. 2A). Also, characteristics of the peptides are given in Table 1. Proton NMR spectroscopy (Fig. 2) performed in *d*_*6*_-DMSO, was used to confirm the chemical structure of these conjugates, demonstrating the presence of peptide via its specific protons, as well as other protons in FA and the PEG polymer. It was clear that proton signals belonged either to an N-terminus modified peptide (C-Mel) or a C-terminus modified peptide (Mel-C) gave a similar peak profile for both conjugates. A comparative representation of the spectra for C-Mel/Mel-C peptides of all conjugates revealed the presence of labile protons (–N*H*_2_ and Ar–*H*), that appear as a broad multiplet between 8.66 ppm and 6.64 ppm. In addition, Melittin's tryptophan amino acid is the source of the aromatic group (Ar–*H*) of peptide protons. The amide (–O=C–N*H)* protons of the peptides as a broad multiplet at 4.00–5.00 ppm and isopropyl group protons of peptides as a doublet at 0.79–0.88 ppm; these may have originated from the structure of the targeting peptide or C-Mel/Mel-C peptides. The presence of heterofunctional linkers was determined by the presence of a peak at 3.50 ppm that belonged to the O–C*H*_2_ protons of the PEG polymer. In FA–containing conjugates, the aromatic (Ar–*H*) and labile (–COO–*H*) protons of FA were observed at 6.64 ppm and 10.76 ppm, respectively. For the other conjugate that had a targeting peptide in its structure, the –COO–*H* protons seem to appear at 10.76 ppm, as well. FT-IR analysis of the conjugates (CM–FA, MC–FA, CM–Target, and MC–Target) and peptides (C-Mel, Mel-C, and the targeting peptide) were able to identify the presence of the FA moiety, peptides, and ethylene glycol units present in the linkers (Fig. 3). The stretching bonds of groups containing labile protons (O–H, –O=C–O–H, and –N–H) were seen at approximately 3280–3544 cm^−1^. For all spectra, regular –C–H and –C–C stretching appears at around 2869–2967 cm^−1^. In addition, the stretching peaks of primary amide carbonyl groups (1636–1650 cm^−1^), –C–O of the PEG polymer (1096–1200 cm^−1^), and aromatic groups (Ar–*H,* 792–843 cm^−1^) were seen for all conjugates, peptides, and FA. However, the stretching peaks of secondary amide carbonyl groups were seen at around 1536–1540 cm^−1^ in all conjugates. The presence of –C–S stretching peaks (947–950 cm^−1^) in the spectra of all conjugates points out that the peptides had successfully bound to the OPSS linker. Furthermore, the –C–N stretching peak seen at 1340 cm^−1^ clearly confirmed FA attachment to the conjugate structure. The breaking of disulfide bonds contained in the linker molecule due to redox reactions is evaluated in Supp. Data Fig. 3. Since the Dithiothreitol (DTT) peak gives its maximum absorbance at 280 nm, the DTT peak was examined alone at 280 nm. Due to the PEG2000 polymer contained in the linker molecule, molecules were observed in the range of 190–200 nm, especially after the linker breakage. It is observed that the linker is broken due to redox events after incubation of the selected conjugate molecule with DTT. After this breakage, the linker molecule attached to folic acid exhibited a hydrophilic behavior, while the peptide molecule was observed in a more hydrophobic region.

**Table 1:** Synthesized peptide characteristics.

Modified peptide	Modified peptide sequence	pI	Net charge at pH 7	Mass (Da)	Hydrophobicity (kcal * mol^−1^)
Mel	GIGAVLKVLTTGLPALISWIKRKRQQ	12.51	5	2845.7331	15.64
C-Mel	CGIGAVLKVLTTGLPALISWIKRKRQQ	11.71	5	2948.7422	15.62
Mel-C	GIGAVLKVLTTGLPALISWIKRKRQQC	11.63	5	2948.7422	15.62
Targeting peptide	CKQNLAEG	6.18	0	861.4002	16.33

**Figure 4:**
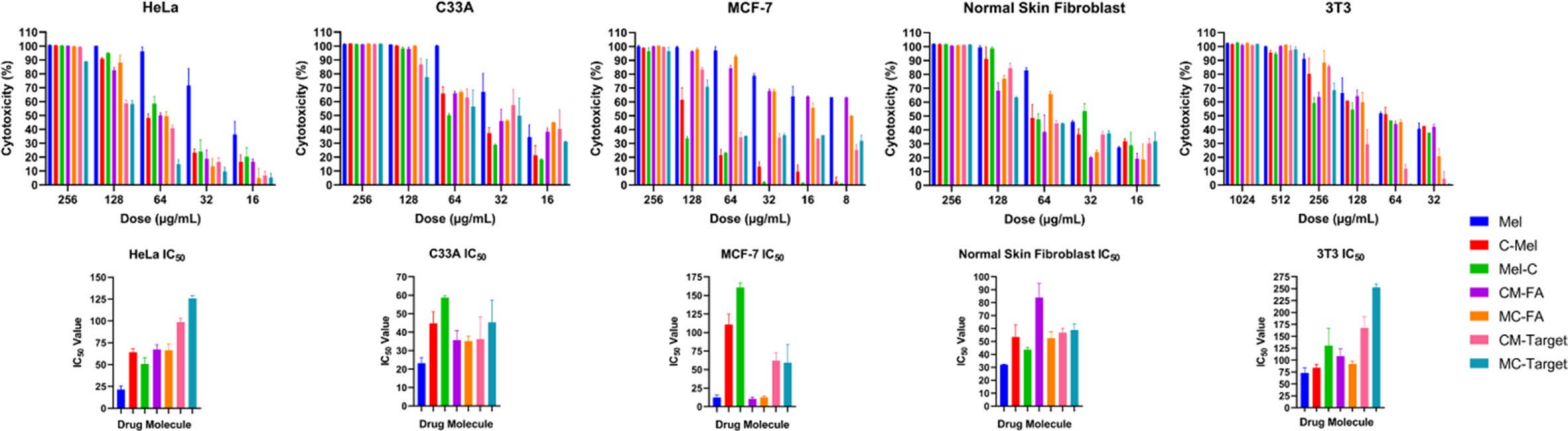
Dose-dependent peptide/conjugate cytotoxicity towards HPV (+) human cervical adenocarcinoma (HeLa), HPV (−) human cervical squamous carcinoma (C33A), human breast cancer (MCF-7), human normal skin fibroblast (NSF), and mouse embryonic fibroblast (3T3) cells for 24 h. The cell lines were treated with the indicated peptides (Mel, C-Mel, and Mel-C) and conjugate (CM–FA, MC–FA, CM–Target, and MC–Target). The targeting peptide alone was tested on cell lines and the peptide was non-toxic (data was not shown). The inhibitory concentration values (IC_50_) were determined using the MTT assay formula (see “Methods”). Data are shown as mean ± SD (three replicates) and were subjected to two-way analysis of variance (ANOVA). Differences in cytotoxicity among peptides and conjugates were found to be statistically significant (p < 0.0001) for all plotted graphs.

An MTT assay was performed to evaluate in vitro cytotoxicity of all peptides and conjugates. It is graphically shown that the determined cytotoxicity increased proportionally with increasing peptide/conjugate dose (Fig. 4). IC_50_ values of the peptide and conjugate molecules were evaluated. According to this, the Mel peptide has the highest cytotoxicity of all molecules in all cell lines tested (except CM–FA_MCF-7_: 10.38 µg/mL). Mel peptide had the highest cytotoxic effect in breast cancer cells (MCF-7: 12.45 µg/mL), whereas mouse fibroblast cells (3T3: 72.95 µg/mL) had the lowest. When the cytotoxicity of Mel peptide was examined in cancer cell lines, it exhibited quite similar cytotoxicity values in cervical cancer cell lines (HeLa: 21.59 µg/mL, C33A: 23.12 µg/mL) but significantly higher cytotoxicity in breast cancer cells. Mel peptide is less cytotoxic in healthy cell lines than cancer cell lines. When healthy cell lines were evaluated, higher cytotoxicity was observed in human skin fibroblasts (NSF: 32.16 µg/mL).

Cysteine versions of Mel show the lowest cytotoxicity at MCF-7 (C-Mel: 110.59 µg/mL, Mel-C: 160.62 µg/mL), while the highest cytotoxicity for C-Mel at C33A (44.76 µg/mL) showed in NSF (43.66 µg/mL) for Mel-C. Other C-Mel and Mel-C values are as follows: HeLa: 64.15 µg/mL, NSF: 53.39 µg/mL, 3T3: 83.99 µg/mL for C-Mel; HeLa: 50.72 µg/mL, C33A: 58.63 µg/mL, 3T3: 130.49 µg/mL for Mel-C.

FA containing–conjugates showed similar cytotoxicity values in all tested cancer lines individually (CM-FA_HeLa_: 67.34 µg/mL, MC–FA_HeLa_: 66.43 µg/mL; CM-FA_C33A_: 35.75 µg/mL, MC-FA_C33A_: 35.22 µg/ mL; CM-FA_MCF-7_: 10.38 µg/mL, MC-FA_MCF-7_: 12.63 µg/mL). The FA containing–conjugates showed the highest cytotoxicity at MCF-7 and the lowest toxicity at 3T3 (CM–FA: 107.97 µg/mL, MC–FA: 91.75 µg/mL). CM–FA shows less cytotoxicity in the NSF cell line than MC–FA (CM–FA: 83.82 µg/mL, MC–FA: 52.44 µg/mL).

Among cervical cancer cell lines, targeting peptide–containing conjugates had the highest cytotoxic effect in C33A, which was approximately 2.7 times more than HeLa (CM–Target_C33A_: 36.20 µg/mL, MC–Target_C33A_: 45.27 µg/mL; CM–Target_HeLa_: 98.87 µg/mL, MC–Target_HeLa_: 125.86 µg/mL). The cytotoxicity values of CM-Target and MC–Target in MCF-7 were 61.89 µg/mL and 59.27 µg/mL, respectively. The cytotoxicity of Targeting peptide-containing conjugates in healthy cell lines was lowest in 3T3 (CM–Target_3T3_: 167.26 µg/mL, MC–Target_3T3_: 252.50 µg/mL; CM–Target_NSF_: 56.73 µg/mL, MC–Target_NSF_: 58.82 µg/mL).

**Figure 5:**
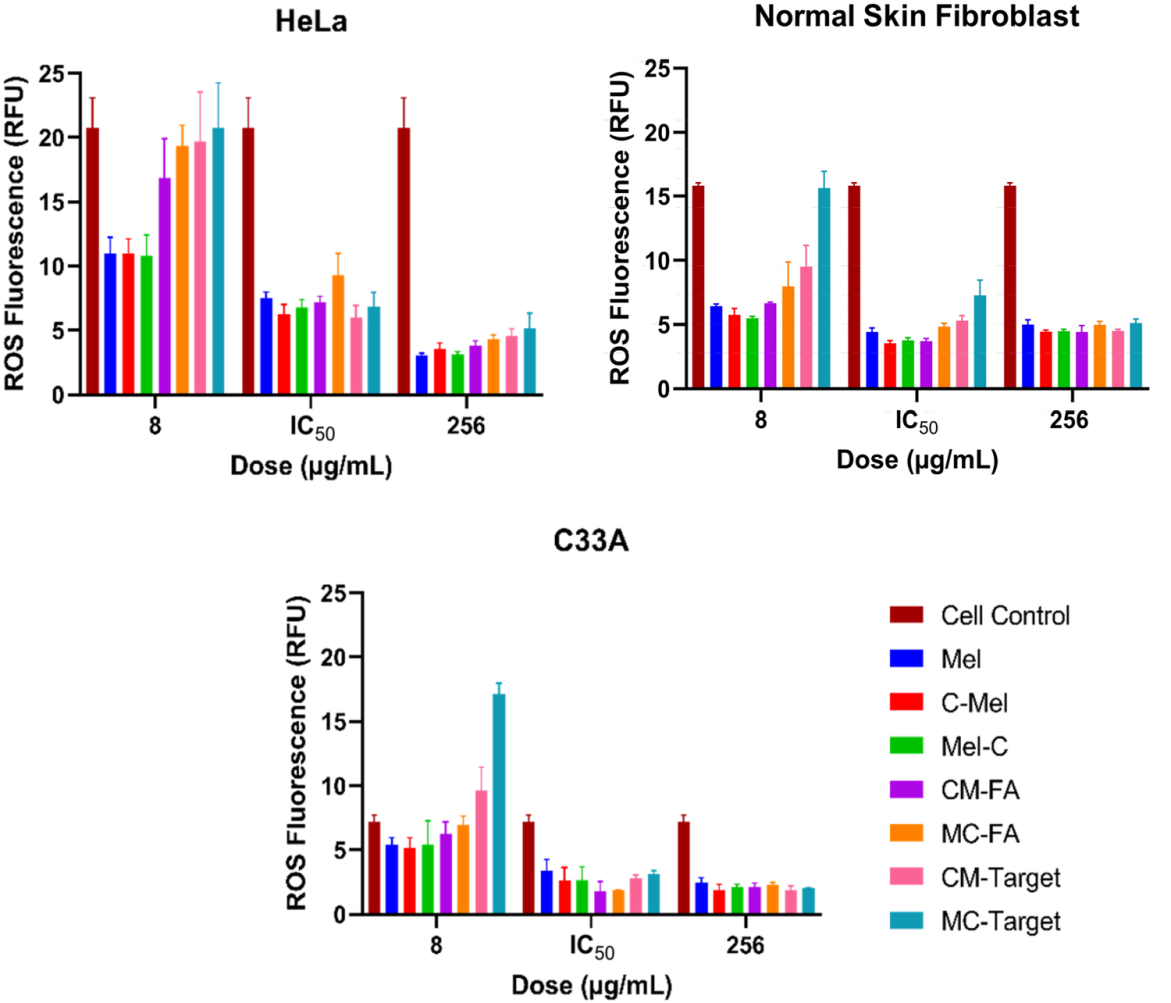
Reactive oxygen species (ROS) determination after treatment with the peptides (Mel, C-Mel, and Mel-C) and conjugates (CM–FA, MC–FA, CM–Target, and MC–Target) on three cell lines (C33A, HeLa, and NSF). Three different drug doses of each peptide and conjugates were tested on the cell lines. According to the cytotoxicity test, the non-toxic dose, 50% toxic dose, and 100% toxic dose were determined to be 8 µg/mL, IC_50_’s, and 256 µg/mL; these were used as the three concentrations for the ROS assay. 200 µM TBHP (positive control) treatment was determined as 51.26 RFU, 36.46 RFU, and 60.70 RFU for HeLa, NSF, and C33A, respectively (data not shown). Using a ROS assay formula (see “Methods”), the RFU values were then calculated. Data are shown as mean ± SD (three replicates) and were subjected to a two-way analysis of variance (ANOVA). Differences in response among peptides and conjugates were statistically significant (p < 0.0001) for all graphs depicted.

Following the evaluation of cytotoxicity, we investigated oxidative stress conditions (Fig. 5). The ROS test was carried out using human cell lines HeLa, C33A, and NSF. The cells' basal ROS levels were given as the cell control when no peptide/conjugate molecule was administered. ROS levels are around 16 RFU in healthy human cell line (NSF), whereas in cancer cell lines, around 21 RFU and 7 RFU in HeLa and C33A, respectively. The concentrations at which all peptide/conjugate molecules were used for the ROS test were determined according to the cytotoxicity results. These concentrations are below 50% cytotoxicity (8 µg/mL), 50% cytotoxicity (IC_50_), and almost 100% cytotoxicity (256 µg/mL). No significant difference was observed in the ROS levels of the equivalent concentrations of the peptides (Mel, C-Mel/Mel-C) administered to each cell line. At IC_50_ and 256 g/mL, the conjugates reduced ROS levels by roughly three-fold in NSF and at least two-fold in cancer cell lines. In contrast, when the conjugates were applied to cell lines at low concentrations (8 µg/mL), they enhanced the ROS level in the cells compared to the peptides. In particular, the MC–Target conjugate increased ROS levels in C33A to much higher levels than cell control. In summary, the administration of IC_50_ values obtained from cytotoxicity results revealed that the conjugates reduced the ROS levels compared to the cell control approximately as much as the Mel peptide.

To obtain MIC values, the synthesized peptides and conjugates were then tested on one of the most common gram-negative and -positive strains, *Escherichia coli* (*E. coli*) and *Staphylococcus aureus* (*S. aureus*), respectively. No inhibitory effects were observed at any of the concentrations tested in the areas marked “X” and bacterial growth was seen (Table 2). Among the peptides studied (targeting peptide, C-Mel/Mel-C, Mel), Mel peptide had the highest antibacterial activity, while MC–FA had the highest activity in all conjugates. The C-Mel and Mel-C peptides showed the same results against both bacteria, and the Mel-C peptide showed twice as much antibacterial activity as the C-Mel peptide. For all conjugates (except CM–FA) we obtained the same results against both bacteria.

**Table 2:** MICs for peptides and conjugates against common gram-positive and gram-negative bacteria.

Drug molecule	Minimum inhibitory concentration (µg/mL)	
	*E. coli* 25922	*S. aureus* 25923
Mel	8	16
C-Mel	64	64
Mel-C	32	32
Targeting peptide	X	X
CM–FA	128	256
MC–FA	64	64
CM–Target	128	128
MC–Target	128	128

The MIC values after the treatment with the plasma proteases of the Mel peptide and conjugates were evaluated from 0 to 24 h time intervals. No inhibitory effects were observed at any of the concentrations tested in the areas marked “X” and “blood plasma” alone was used as the control group (Table 3). The biological activity of the Mel peptide was stable from 0 to 6 h at 15.6 µg/mL and increased by two-fold at 24 h. The Mel-C containing conjugates showed similar patterns of biological activity, as their MIC values decreased two-fold from 6 to 24 h. In contrast, the biological activity of CM–FA doubled after 30 min (from 62.5 to 125 µg/mL), while CM–Target was stable for all time points (62.5 µg/mL). Also, the bacterial growth was observed in only blood plasma as expected.

**Table 3:** Biological activity and stability of the peptide and conjugates following incubations with plasma proteases.

Drug molecule/time (h)	MIC after plasma proteases (µg/mL)			
	0 h	0.5 h	6 h	24 h
Mel	15.6	15.6	15.6	7.8
CM–FA	62.5	62.5	125	125
MC–FA	31.25	31.25	31.25	62.5
CM–Target	62.5	62.5	62.5	62.5
MC–Target	62.5	62.5	62.5	125
Blood plasma	X	X	X	X

**Figure 6:**
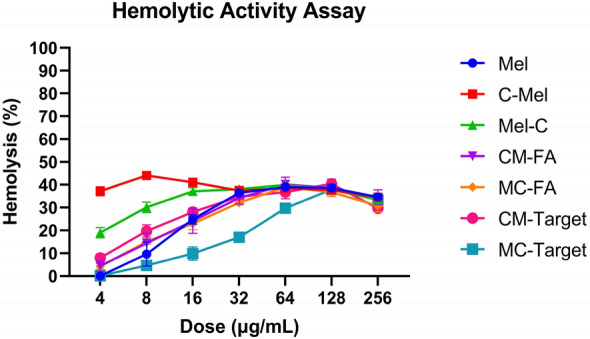
Dose-dependent hemotoxicity of peptides (Mel, C-Mel, and Mel-C) and conjugates (CM–FA, MC–FA, CM–Target, and MC–Target) against human red blood cells. Data are shown as mean ± SD (three replicates) and were subjected to a two-way analysis of variance (ANOVA). These differences were found to be statistically significant (p < 0.0001).

After stability test in blood plasma, we investigated the hemolytic activity of synthesized peptides and conjugates on human erythrocytes (Fig. 6). In general, hemolytic activity of peptide/conjugates increased in a dose-dependent manner. We found that the C-Mel peptide showed the highest hemotoxicity, while MC–Target had the lowest hemotoxicity.

**Figure 7:**
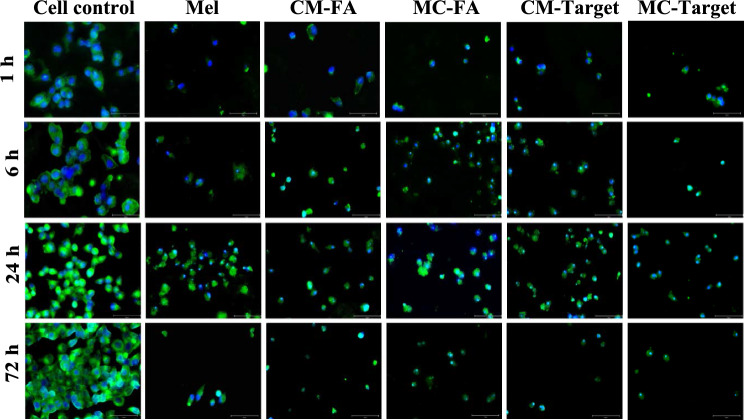
Fluorescent images taken of HeLa cells following treatments with IC_50_’s of the Mel peptide and conjugates (CM–FA, MC–FA, CM–Target, and MC–Target) at different time intervals (1 h, 6 h, 24 h, and 72 h). Alexa Fluor 488 (green) and DAPI (blue) show the mammalian cell membrane and nucleus, respectively (scale bars indicate 75 µm).

The IC_50_ values of the Mel peptide and the conjugates on HeLa cells were observed by fluorescent microscopy at different time intervals (Fig. 7). At time intervals from 1 to 72 h, we observed a time-dependent increase in the cell control, while in general we observed a decrease in cell viability after the treatment with all peptides and conjugates relative to cell control. Moreover, we found HeLa cell controls preserved their morphological structure at all time intervals. After HeLa cells treated with peptides and conjugates, we saw a loss of membrane integrity that resulted in morphological distortion of the cells. Furthermore, after the treatment with peptide/conjugates, intracellular compartments leak out full or partially, forming clear zones inside the cell, whereas cell nuclei retain their shape.

## Discussion

With today’s technology, it has been revealed that peptides derived from nature may be used in cancer treatment^20^. Melittin, with a + 5 charge, is a biologically active peptide found in bee venom^21^. Melittin's charge causes it to bind to cancer cell membranes and damage them through its mechanism^22,23^. This characteristic enables it to be used in future anticancer therapies.

**Figure 8:**
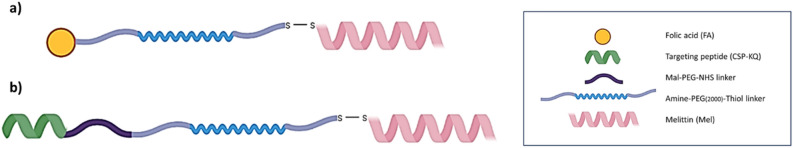
Illustration of the synthesized conjugates. (**a**) FA–containing conjugates, (**b**) targeting peptide–containing conjugates.

Recently, the development of targeted cancer treatments has helped to ensure that patients are exposed to fewer side effects, thereby reducing the negative impact of cancer treatment on well-being^24,25^. In this study, we created two main conjugate designs by attaching two different targeting agents, FA and squamous cell carcinoma-specific peptide^15^, to the Melittin peptide (Fig. 8). Each primary design contained two sub-designs in which the Melittin peptide was bound to either the N- or C-terminus. All designs considered the features of the cancer microenvironment, and we therefore created a novel redox-responsive cargo system tailored for delivery (Supp. Data Fig. 3). Next, we tested the cytotoxicity, ROS activity, hemolytic activity, protease activity, and antibacterial properties of the anticancer conjugates in vitro.

We then evaluated the effectiveness of our anticancer conjugates against various cell lines to be candidates for novel targeted medications (Fig. 4). In experiments to examine the cytotoxic properties of the conjugates, the cytotoxicity of the Mel peptide alone is highest in all cell lines (except MCF-7). The results show that the cysteine added Mel peptides are less toxic than Mel alone. As a binding strategy, adding a cysteine residue to the -N or -C terminus of the peptide may have influenced how Melittin interacts with cell membranes and its overall cytolytic activity. Because it has been proven that thiol-containing peptides interact with thiol groups on cell surfaces and can affect membrane proteins were either trapped or internalized^13^. Due to redox reactions, C-Mel/Mel-C peptides are expected to be released in the cancer microenvironment by breaking the disulfide bonds in the conjugates. In this case, it is crucial to determine the cytotoxicity of C-Mel/Mel-C peptides alone. The decrease in cytotoxic activities of thiolated Mel peptides compared to the Mel peptide may have occurred due to the change in how thiol groups interact with cell membranes. Mel peptide alone was more cytotoxic in MCF-7 cells than cervical cancer cell lines. However, the C-Mel/Mel-C versions have the lowest cytotoxic activity in cell lines compared to Mel peptide. When thiolated peptides were coupled with FA, their cytotoxic activity increased drastically. This increase is due to the fact that MCF-7 cells are FR positive cell lines^26^. Breast cancer cells contain 100–300 times more FRs than healthy cells^14,27^. Since FR is overexpressed in many cancers including lung, breast, and cervical, it holds great potential for targeted cancer therapy^28,29^. Targeting peptide–containing conjugates have much less cytotoxicity in MCF-7 cells than FA–containing conjugates. Because the targeting peptide is specific for squamous cervical cancer^15^. Furthermore, the higher cytotoxic activity of targeting peptide–containing conjugates compared to C-Mel/Mel-C can only be explained by the linker molecule being specific to the cancer microenvironment.

Mel peptide showed much higher cytotoxic activity than all the conjugates except CM–FA_MCF-7_. This might be because polymeric molecules in the linker, such as PEG, mask or circulate around the peptide molecules, limiting their activity. On the other hand, the use of these polymers can be advantageous with their properties such as increasing solubility and stability, and decreasing hemolytic activity^30^. Moreover, future in vivo tests will provide a better understanding of the conjugates' real targeting effect.

After evaluating the conjugates on C33A and HeLa cells, all conjugates were found to have a significantly higher cytotoxic effect in C33A than HeLa. Especially targeting peptide–containing conjugates have higher activity in C33A due to their squamous cell carcinoma specificity. In addition, mouse fibroblast and human fibroblast cell lines were studied to understand the cytotoxic effect of peptide/conjugate in healthy cells. Mouse fibroblasts were used to lead future animal trials, while human fibroblast cells were used for comparison with human cancer cell lines. An attempt has been made to determine IC_50_ concentrations of conjugates that can kill cancer cells without killing healthy cells. Some findings in this range are as follows: While FA–containing conjugates can be used in both C33A and MCF-7, targeting peptide–containing conjugates can only be used in C33A.

When the advantages of conjugates regarding cytotoxicity are considered, many cancer drugs currently on the market are cytotoxic against both healthy and cancer cells^31,32^. Since our conjugates contain molecules such as modified peptides, targeting agents, and polymers, their cytotoxicity against healthy cells is much less. Our aim is to increase cytotoxicity as much as possible for cancer cells while decreasing it for healthy cells. In particular, while the CM–FA conjugate further enhances the cytotoxic effect of the peptide on breast cancer, it provides a significant benefit that it reduces this effect in healthy cell lines.

The ROS test results revealed a significant decrease in the RFU levels of cells following the administration of peptide and conjugate molecules relative to cell control (except 8 µg/mL) (Fig. 5). This decrease suggests that the Melittin peptide and its conjugates may also have antioxidant activity as well as anticancer effects. Interestingly, conjugates can also have the opposite effect when administered at a value less than IC_50_. Moreover, our results are supported by other recent studies, which also suggest that Melittin has antioxidant and anti-inflammatory properties^33,34^. On the molecular level, high ROS activity affect cells by attacking molecules such as DNA, proteins, and lipids. This causes DNA damage, protein oxidation, and lipid peroxidation in the cell, resulting in genetic changes that cause cancer^35^.

Afterward, the antibacterial activities of the conjugates were investigated. Mel peptide, which has anticancer properties, is also known to have antibacterial properties^36^. We used the most prevalent gram-negative and positive bacteria to see if this antibacterial activity was also present in the conjugate forms for the MIC test (Table 2). According to the findings, the addition of cysteine and targeting agents to the Mel peptide considerably reduces the antibacterial activity of the Mel peptide. However, this decrease is less in the Mel-C peptide compared to the C-Mel peptide. In other words, Mel's N-terminus reveals more antibacterial activity compared to its C-terminus.

When the properties of the Mel peptide are examined, the first 20 amino acids provide membrane activity, while the 12–22 residues causes lytic/antimicrobial activity^37^. The finding that the final six amino acid sequences have no influence on activity may be helpful in demonstrating that the N-terminus has a more effect on the antibacterial property.

In addition, no correlation was found between the anticancer and antimicrobial activity. One example is that the targeting peptide, one of the targeting agents, does not target bacteria but can target cervical cancer cells. Since the tested bacteria do not have a folic acid receptor or a system to metabolize folic acid directly, the targeting agent FA also showed no positive/negative effect to bacteria^38,39^. The PEG polymer in the linker molecule might explain why the conjugates have even lower antibacterial activity than the cysteine peptides because our previous study has shown that in polymer–antimicrobial peptide conjugates, the PEG polymer can surround the peptide and reduce its activity^30^.

The significant advantage of performing a plasma stability test with plasma proteases is to observe how long drug molecules can remain in the bloodstream without losing their biological activity (Table 3). We also think that this experiment may provide useful information for future in vivo experiments^40^.

Biological effects of proteases were observed due to incubation with *E. coli* after plasma proteases acted on peptide/conjugate molecules. When the MIC test findings with *E. coli* are compared, they almost identically match the plasma protease data after 24 h. The inhibitory concentrations determined for *E. coli* in the MIC and plasma protease assay were correlated.

Microbial proteases can be secreted by bacteria^41^. The plasma protease test involves incubating plasma proteases and peptides/conjugates for 24 h before adding a protease inhibitor. Although the bacteria added to the test as a last step and they secrete any microbial protease, it is hypothesized that the protease inhibitor inactivates them. In conclusion, it has been observed that peptide/conjugates incubated with plasma proteases for 24 h (MIC after plasma proteases assay) have very similar effects to peptides/conjugates incubated with microbial proteases for 24 h (MIC assay).

When the stability of targeting peptide–containing conjugates is compared to that of FA–containing conjugates, the targeting peptide–containing conjugates seem to be more stable. One of the reasons for this may be that, since the targeting agent is a kind of peptide, the proteases are actually able to cleave not only the Mel peptide but also the targeting peptide. It was assumed that the stability duration of the Mel peptide might be extended since the tendency of the proteases will be on both peptides. Another reason may be that by binding to its membrane-active segment at the N-terminus of the Mel peptide, it will be more difficult for proteases to attack the CM–Target conjugate; thus, it may have remained more stable than the other conjugates.

To understand the effect of the peptides and conjugates on human red blood cells, the hemolytic activity assay was conducted for 1 h. As shown in Fig. 6, all tested molecules were below the HC_50_ value (HC_50_, representing 50% hemolysis). When the IC_50_ values of the peptide/conjugate molecules were used, the molecules at these concentrations were indicated as hemotoxic since the HC_5_ (HC_5_, representing 5% hemolysis) value was higher than the non-hemotoxic standard value^42^.

Considering the hemolytic values, topical application may be more appropriate instead of intravenous injection. Intravaginal topical application has many advantages, such as easy access to the cervix, localized targeting of squamous cell carcinoma without damaging healthy tissues, and increasing diffusion of the low MW PEG molecule in the conjugates in the vaginal mucosa^43^. In addition, since the vaginal pH is in the 3.8–5 range^44^, release of peptides will be much easier.

Melittin peptide, with a + 5 charge, has an affinity and can attach to the negatively charged membrane of cancer cells. The peptide then pulls the membrane electrostatically, causing the formation of pores. Previous studies have demonstrated the effectiveness of the pore formation mechanism of similar alpha-helical structure-forming peptides^45^. As a result of pore formation, cells become unviable since water and hydrophilic molecules leak out of the cell. Moreover, Melittin peptides cooperate among themselves, causing conformational changes to the membrane due to deformation and membrane lipid release^46^. Melittin also causes different effects on the membrane according to the dose administered to the cell. The peptide molecule, which is effective even at nanomolar doses, causes the formation of temporary pores that allow ions to pass through the cell membrane, but not monosaccharides, or larger molecules. However, at micromolar levels, it creates stable pores that allow the passage of molecules up to tens of kilodaltons^22,23,47^. The effects of the mechanism by which the Melittin peptide damages the cells by forming pore can be seen in Fig. 7. Since it was obvious that the synthesized conjugates showed the best effect on C33A cells, we wanted to observe their effects on HeLa cells under a microscope. While the growth of the control cells increased from 1 to 72 h, we observed a decrease in growth due to cytotoxic effects when the cells treated with the peptides and conjugates (Fig. 4). Moreover, peptides and conjugates could show an anticancer effect even at the first hour.

## Conclusions

In conclusion, the conjugates designed here have been shown to have both anticancer and antibacterial activity as revealed by in vitro tests. The stable CM–Target conjugate, which is specially designed for cervical squamous cell carcinoma, is a promising targeted treatment agent. Interestingly, FA–containing conjugates were highly efficient in all tested cancer cell lines, especially in breast cancer. The conjugates also exhibit lower cytotoxicity on healthy cells than the peptide alone. All conjugates had a significant antioxidant impact at their corresponding IC_50_ values and 256 µg/mL. In addition, while cysteine added peptides alone had higher hemolytic activity, the conjugates created from these peptides showed less hemotoxicity. Our results suggest that anticancer conjugates developed here represent promising future drug candidates, providing more targeted therapies compared to natural peptides.

## Materials

Anhydrous Dimethylformamide (DMF), dimethyl sulfoxide (DMSO), folic acid (FA), N-(3-Dimethylaminopropyl)-N′-ethyl carbodiimide hydrochloride (EDCI), triethylamine (Et_3_N), N-Hydroxysuccinimide (NHS), diethyl ether, Paraformaldehyde (PFA), methanol (MeOH), diisopropylcarbodiimide (DIC), oxyma, trifluoro acetic acid (TFA) were purchased from Sigma-Aldrich (USA). Dulbecco’s modified Eagle’s medium (DMEM, Gibco), Eagle’s minimal essential medium (EMEM, Gibco), fetal bovine serum (FBS, Gibco), penicillin–streptomycin antibiotic mixture (Pen/Strep, Sigma Aldrich), phosphate buffered saline (PBS), DAPI (Thermo Fisher), and AlexaFluor488 (concanavalin A, Thermo Fisher) were used for mammalian cellular assays. *Escherichia coli* (*E. coli*, ATTC 25922) and *Staphylococcus aureus* (*S. aureus*, ATTC 25923) were used for bacterial cell culture experiments. Bacteria were grown in Muller Hinton Broth (MHB, Oxoid, UK) and Muller Hinton Agar (MHA, Oxoid, UK). For other in vitro assays, triton X-100 (Sigma-Aldrich) and a protease inhibitor (cOmplete, Roche) were used.

## Methods

### Peptide synthesis

All peptides listed in Table 1 were synthesized using the standard Fmoc-based solid-phase synthesis method on a peptide synthesizer (CEM, Liberty™ Blue). Synthesis was performed on ring amide resin (0.7 mmol/g). DIC and oxyma were used as coupling agents and piperidine was used as a deprotection agent. For cleavage, the peptide was subjected to a TFA/ddH_2_O/triisopropylsilane (i.e., 4750 µL, 125 µL, and 125 µL) mixture for 30 min at 37 °C using a Razor (CEM). The naked peptide was then obtained by diethyl ether extraction at − 20 °C, overnight. Next day, the solution was centrifuged at 6000 rpm for 3 min three times, and pellet was dried under a high vacuum.

### Conjugate synthesis

Next, we synthesized an N-terminus cysteine modified-Melittin–folic acid functionalized conjugate (CM–FA) and a C-terminus cysteine modified-Melittin–folic acid functionalized conjugate (MC–FA). To synthesize these conjugates, in the first step, folic acid (FA, Sigma Aldrich, 2.8 mg, 0.0063 mmol), EDCI (1.48 mg, 0.0095 mmol), Et_3_N (1.2 mg, 0.0119 mmol), and NHS (1.08 mg, 0.0094 mmol) were added to 1 mL DMF at room temperature (RT) for 1 h in the dark. In the second step, an OPSS-PEG-NH_2_ linker (Biopharma PEG, 12 mg, 0.006 mmol) was dissolved in 2 mL DMF. The FA solution and the linker solution were then mixed at RT for 4 h in the dark. Once the FA–linker reaction was complete, dissolved C-Mel or Mel-C peptides (18.6 mg, 0.0063 mmol) in 2 mL DMF were added to the FA–linker mixture and left at RT for 48 h in the dark. After 48 h, the reaction mixture was precipitated in 30 mL cold diethyl ether at − 20 °C, overnight. Then, the mixture was centrifuged at 6000 rpm for 10 min, and the pellet was dried by vacuum. All reactions occurred in an inert environment.

To synthesize an N-terminus cysteine modified-Melittin–targeting peptide-functionalized conjugate (CM–Target) and a C-terminus cysteine modified-Melittin–targeting peptide-functionalized conjugate (MC–Target), in the first phase, Mal-PEG_6_-NHS ester (Sigma Aldrich, 15 mg, 0.0250 mmol) linker, a targeting peptide (25.74 mg, 0.0299 mmol) and Et_3_N (2.52 mg, 0.0249 mmol) were added to 3 mL DMF. This reaction mixture was left at RT overnight. Two round bottom flasks (rbfs) containing the same chemicals except modified Mel peptides were used for CM–Target and MC–Target reactions. In the second phase, an OPSS-PEG-NH_2_ linker (Biopharma PEG, 15 mg, 0.0075 mmol), C-Mel or Mel-C peptide (26.55 mg, 0.009 mmol) and Et_3_N (2.52 mg, 0.0249 mmol) were mixed in 3 mL DMF at RT, overnight in the dark. For both C-Mel and Mel-C, this reaction was performed in two separate rbfs. In the third phase, the second reaction products were added to the first reaction products for each conjugate and kept at RT, overnight. Finally, the reaction mixture was precipitated in 30 mL cold diethyl ether at − 20 °C, overnight. Then, the mixture was centrifuged at 6000 rpm for 10 min, and the pellet was dried by vacuum. All reactions occurred in an inert environment.

### Peptide/conjugate purification and characterization

The peptide/conjugate was purificated and analyzed (data not shown) using HPLC (Agilent Technologies, USA) with C-18 column (RPC 250 × 10 mm ID hydrophobic 6 µm, Agilent VariTide). For the CM–FA and MC–FA conjugates’ purification, we used a 0–100% ACN (0.025% TFA) gradient for 30 min, and for the CM–Target, and MC–Target conjugates, we used a 0–100% ACN (0.025% TFA) gradient for 40 min. Peptide methods for HPLC purification are provided in Supp. Data 1. A Nicolet iS10 FT-IR was used to obtain infrared spectra of the peptide/conjugates in the range of 4000–500 cm^−1^. The chemical structure of the peptide/conjugates was characterized by proton nuclear resonance (^1^H NMR) spectroscopy using a 400-MHz Burker NMR spectrometer in DMSO-*d*_*6*_. To evaluate the molecular weight of the synthesized peptide, LC–MS/MS (6420 Triple Quad, Agilent Technologies) was used.

### Cytotoxicity assay

C-33A (HTB-31™, ATCC), HeLa (HTB-31™, ATCC), 3T3 (CRL-1658™, ATCC), Normal Skin Fibroblast (CRL-2091™, ATCC) and MCF-7 (HTB-22™, ATCC) cell lines were cultured in complete EMEM, DMEM, DMEM, EMEM, and EMEM (containing 1% Pen/Strep antibiotic and 10% FBS), respectively. Cells from the different lines were seeded at a density of 5 × 10^4^ cells/well in 100 µL medium placed in a flat-bottom 96-well plate and incubated at 5% CO_2_ and 37 °C. After 24 h, the medium was discarded from the 96-well plate. Serial dilutions of peptide/conjugate (i.e., 256–1 µg/mL) were prepared in triplicate in 100 µL complete medium then added onto each well and incubated at 5% CO_2_ and 37 °C for 24 h. The cytotoxicity was determined by MTT test (MTT, Cell Proliferation Kit, Roche) and measured at 550–690 nm on a microplate reader. The cell cytotoxicity was calculated with respect to control groups treated with only complete medium.

### ROS assay

To evaluate the ROS activity of peptide/conjugates, a ROS kit (DCFDA/H2DCFDA—Cellular ROS Assay Kit, Abcam) was applied to C33A, HeLa, and Normal Skin Human Fibroblast cells. 25,000 cells/well were seeded on a flat-bottom 96-well plate and incubated at 37 °C and 5% CO_2_. After 24 h, the media was discarded, and the wells were washed with 1 × buffer. DCFDA was then added to wells (except for non-stained control cells) and all plates were incubated for 45 min at 37 °C and 5% CO_2_ in the dark. After incubation, peptide/conjugates (Mel, CM–FA, MC–FA, CM–Target, and MC–Target) were added at three different concentrations (i.e., 8 µg/mL, IC_50_ values, and 256 µg/mL) for 5 h. A positive control (TBHP) was also applied. The results were assessed using a microplate reader (Varioskan Flash, Thermo Scientific) at Ex/Em: 485/535 nm. The negative control (non-stained cells) was subtracted from the cell control and drug samples for calculation.

### Hemolytic activity

In this study, human blood cells were taken from healthy donors. This study was approved by Acibadem University Medical Research Ethics Committee (ATADEK). The study was carried out in accordance with the principles of the Helsinki Declaration and the research ethics guidelines of Acibadem University Medical Research Ethics Committee (ATADEK) (Approval No. 2023-2/32; 27 January 2023). Also, the protocols which are hemolytic activity assay, and stability in the presence of plasma proteases' assay were approved by the university ethics committee. Before drawing blood, all donors provided written informed consent.

30 µL of fresh human blood was added to 10 mL of autoclaved tris-saline (10 mM tris, 150 mM NaCl, pH 7.2) and centrifuged at 1500 rpm for 5 min three times. 100 µL of the blood mix was added to each well of a round-bottom 96-well plate. Concentrations of freshly prepared peptide/conjugate (i.e., ranging from 256–4 µg/mL) in tris-saline (100 µL of total volume) were added to the 96-well plate containing the blood mix and the plate kept at 37 °C for 1 h. Triton X-100 in 10% DMSO-treated blood mix and blood mix without addition were used as positive and negative controls, respectively. Next, the plate was centrifuged for 10 min at 1500 rpm. The supernatants were taken into a new round-bottom 96-well plate and measured at 414 nm using a microplate reader^48^. All samples were used as a triplicate.

### Minimum inhibitory concentration (MIC)

The MIC of the conjugates on *E. coli* and *S. aureus* were determined. 95 µL of the MHB containing serial two-fold dilutions of each peptide/conjugate were added to a round-bottom 96-well plate from a maximum concentration of 1024 µg/mL to 0.5 µg/mL. Bacterial samples were taken from an exponentially growing culture in MHB and adjusted to 1.5 × 10^6^ CFU/mL. 5 µL of the prepared bacterial suspension was added to each well and incubated at 37 °C, overnight. The bacterial growth was measured using a microplate reader (Gen5 Synergy HT, Biotek) at 600 nm. Broth containing only bacteria was used as a positive control while broth without bacteria was used as a negative control.

### Stability in the presence of plasma proteases

To separate human plasma from blood, EDTA-treated blood samples taken from healthy subjects were centrifuged for 5 min at 1500 rpm and the supernatant was kept. The peptide/conjugate was added to the human plasma to adjusted to 0.1 mg/mL (2048 µg/mL in 1.7 µL sample completed with plasma) and incubated at 37 °C, 1500 rpm in a shaker. Sample aliquots were taken after 0 h, 30 min, 6 h, and 24 h incubation and kept at − 20 °C^41^. Then, 5 µL of a protease inhibitor was added to 35 µL aliquots of each sample. *E. coli* was incubated in MHA at 37 °C, overnight. Then, the fresh colony was inoculated into MHB and adjusted to 1.5 × 10^8^ CFU/mL (0.5 McFarland). This bacterial solution was diluted with MHB to a ratio of 1:200. Serial dilutions of aliquots (500–0.25 µg/mL) were prepared in MHB (40 µL of total volume) in a round-bottom 96-well plate. The blood plasma was used as a negative control group. 5 µL bacterial solution was added to dilutions and the control group and incubated at 37 °C, overnight^30^. All samples and the control group were prepared in triplicate, and samples were measured at 600 nm using a microplate reader.

### Redox-responsive linker breaking

A degradation experiment was performed to understand the breakage of the linker molecule in the conjugates due to the redox reaction. 200 mM DTT (Thermo Fisher) solution was prepared and mixed with 2 mg/mL conjugate at a ratio of 1:1. As a control group, it was prepared by mixing 1 × PBS with 2 mg/mL selected conjugate at a 1:1 ratio. These two mixtures were kept in a shaker at 37 °C under the same conditions for 24 h, and then analyzed using HPLC. For the analysis, we used a 0%–100% ACN (0.025% TFA) gradient for 11 min.

### Fluorescence microscopy

50,000 HeLa cells/well were seeded on a flat-bottom 96-well plate and incubated at 37 °C, 5% CO_2_ for 24 h. The medium was discarded, and cells were treated with the IC_50_ values of peptide/conjugate (Mel, CM–FA, MC–FA, CM–Target, and MC–Target). After 24 h, the medium was removed, the cells were washed with PBS buffer, shaken gently, then incubated at RT for 5 min. Then, 200 µL of cold 4% PFA was added to each well and incubated at RT for 10 min. PFA was removed slowly. The wells were washed with double ionized water and 200 µL of cold MeOH was added to each well. The plate was left to sit at RT for 5 min. MeOH was removed and all wells were dried with a high vacuum.

For the staining of the cells, the cells were washed 1–3 times with PBS. Then, 20 µg/mL of Concavalin AlexaFluor488 solution was used to stain the membrane of the cells and cells were incubated for 1 h in dark. After that, the stain solution was removed and washed 2–3 times with PBS. Subsequently, 10 µg/mL DAPI solution was used to stain the cell nucleus, and cells were incubated for 1–5 min in dark. The stain solution was removed, and the cells were washed 2–3 times with PBS. The cells were imaged using Fluorescence Microscope (EVOS M5000, Thermo Fisher).

### Statistics

GraphPad Prism 9 was used to analyze data from the ROS, hemolytic and cytotoxicity assays. The data was reported as mean ± SD. Statistical comparisons between dosages and groups were reported using a two-way analysis of variance (ANOVA) with a mixed-effects model. *p*-values less than 0.05 were assessed as statistically significant.

### Ethics approval

The study was performed according to the research ethics guidelines of the Acibadem University Medical Research Ethics Committee (ATADEK, decision no: 2023-2/32).

### Supplementary Information

Supplementary Information.

## Data Availability

All data created or analyzed during this research are included in this published article [and its supplementary information files].
