# Supplementary material for: Development and pharmaceutical investigation of novel cervical cancer-targeting and redox-responsive melittin conjugates
Source: Sci Rep. 2023 Oct 25;13:18225. doi: 10.1038/s41598-023-45537-x (PMC10600185; doi:10.1038/s41598-023-45537-x)
Supplement: Supplementary file 1 — Supplementary Information. [file 41598_2023_45537_MOESM1_ESM.docx]

**Supporting Information for**

**Development of novel cervical cancer-targeting and redox-responsive melittin conjugates and their pharmaceutical investigation**

Seray Sahsuvar^1*^, Rabia Guner ^2*^, Ozgul Gok^3#^, Ozge Can^3#^

1 Department of Medical Biotechnology, Institute of Health Sciences, Acibadem Mehmet Ali Aydinlar University, Istanbul, Turkey

2 Department of Biomedical Engineering, Graduate School of Natural and Applied Sciences, Acibadem Mehmet Ali Aydinlar University, Istanbul, Turkey

3 Department of Biomedical Engineering, Faculty of Engineering and Natural Sciences, Acibadem Mehmet Ali Aydinlar University, Istanbul, Turkey

**HPLC Chromatogram of the Peptides During Purification**

The synthesized peptides were purified using HPLC with C-18 column, for the Mel peptide, 0%–100% ACN (0.025% TFA) for 30 min, for C-Mel and Mel-C peptides, 0%–60% for 1 min, 60%–80% for 10 min, 80%–100% for 3 min, for targeting peptide, 0%–35% for 2 min, 35%–55% for 10 min, 55–100% for 3 min. The Mel peptide was obtained at 63%–72% ACN. In both C-Mel and Mel-C peptides, peptide peaks were obtained at 77%­–79% ACN, and for the targeting peptide, peak was collected at 53%­–57% ACN. Finally, the purity of the collected peaks was analyzed by HPLC (Peak purity: >95, data not shown) and the collected peaks from the peptides were used to synthesize the conjugates.


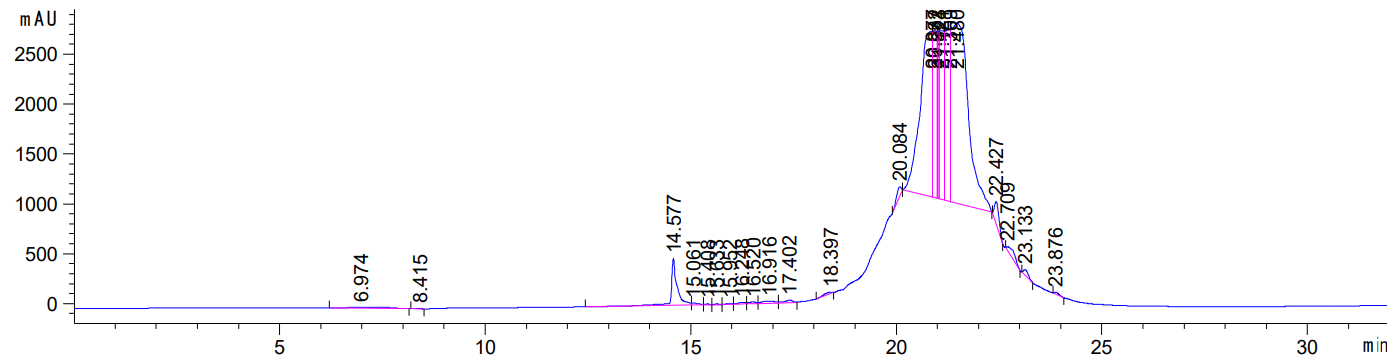
**a)**


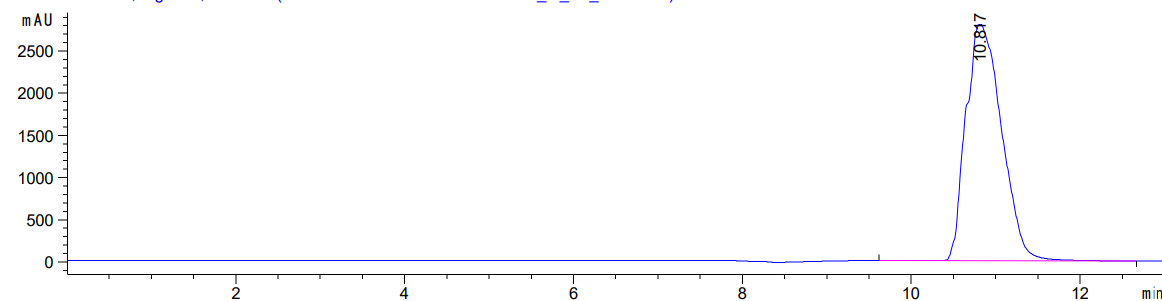


**b)**


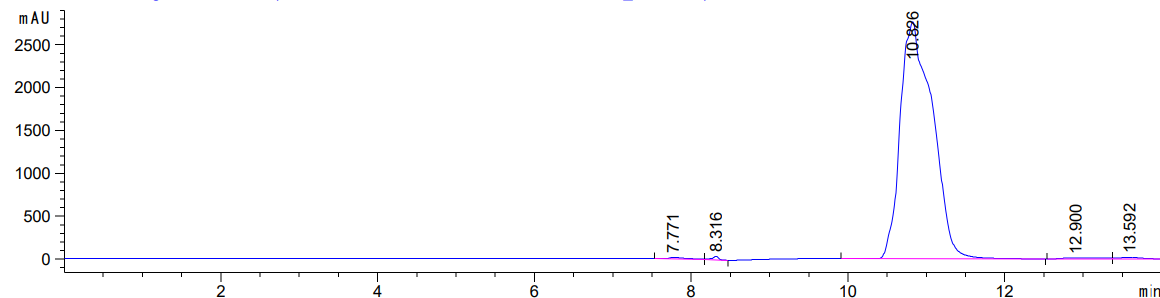


**c)**


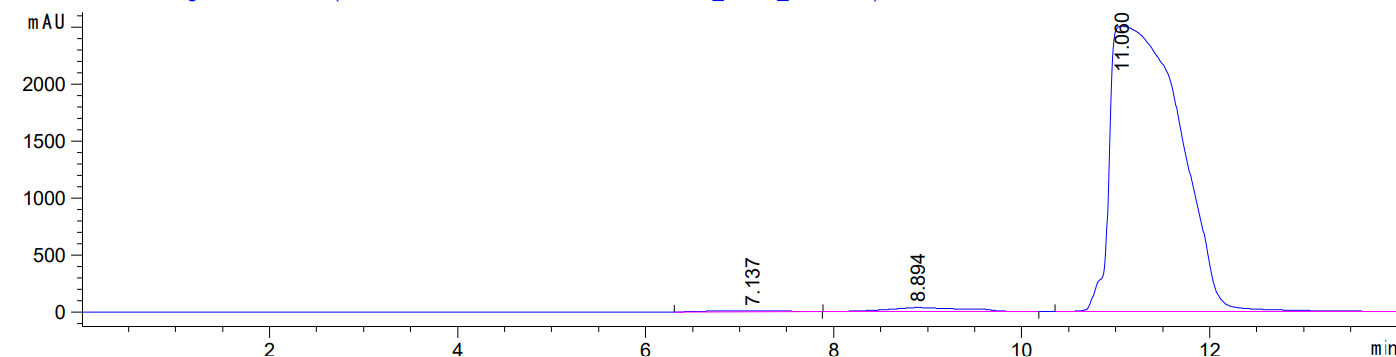


**d)**

**Figure 1.** Semi-preparative HPLC chromatogram of the peptides during purification shown as a) Mel, b) C-Mel, c) Mel-C, d) Targeting peptide.

**Table 1.** Summary of the ACN gradients obtained from HPLC chromatogram of the purified peptide/conjugates.

| Peptide/Conjugate name | HPLC gradient (ACN %) |
| --- | --- |
| Mel | 63–72% |
| C-Mel | 77–79% |
| Mel-C | 77–79% |
| Targeting Peptide | 53–57% |
| CM–FA | 60–80% |
| MC–FA | 60–80% |
| CM–Target | 58–70% |
| MC–Target | 58–70% |

**LC-MS/MS Results of the Peptides**

**a)**

**
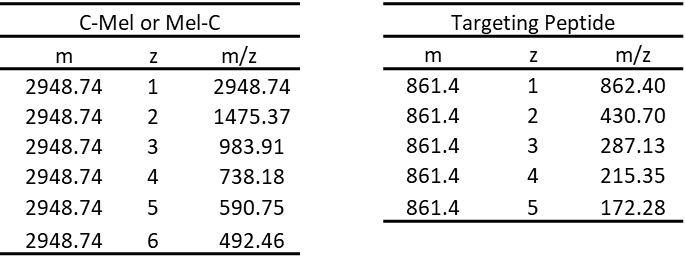
**

**b)**


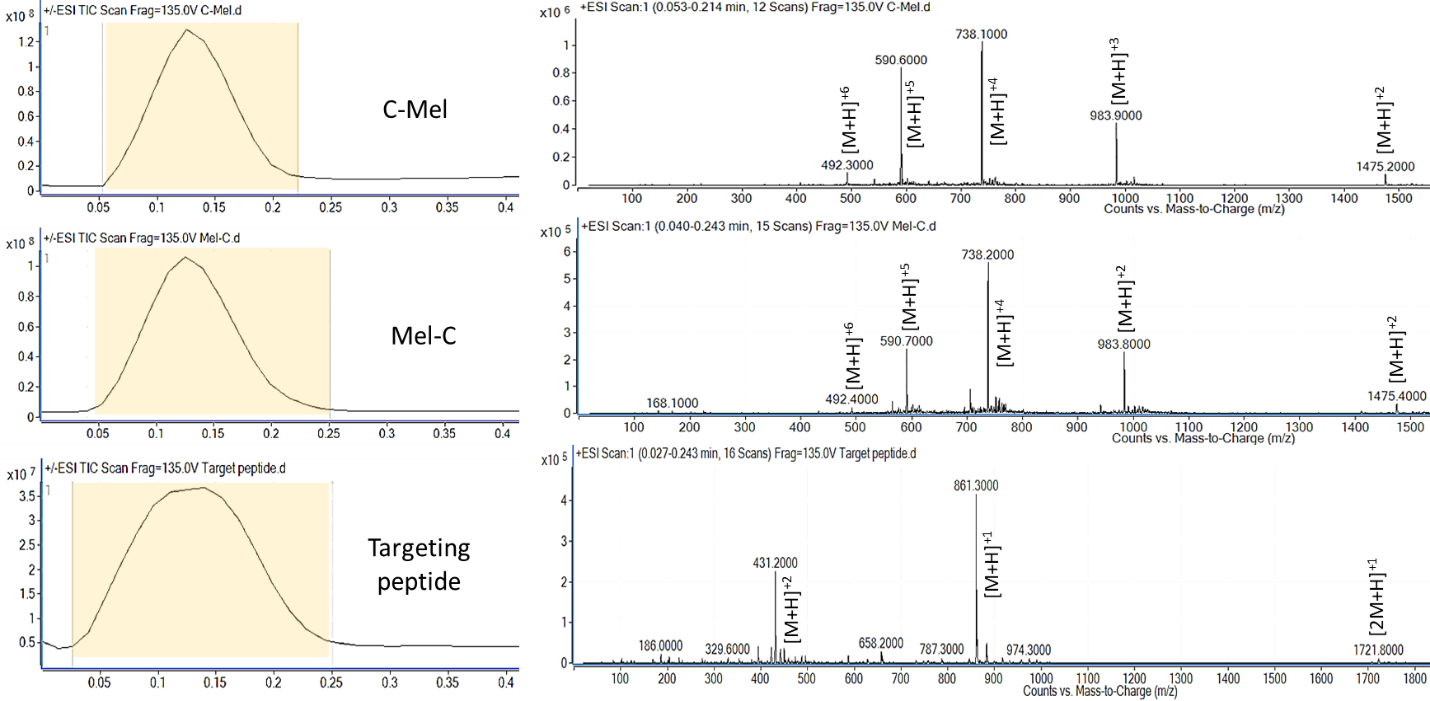


**Figure 2.** LC-MS/MS spectra of the peptides used for conjugation. a) mass to charge ratios of the peptides, b) LC-MS/MS spectra of the peptides.


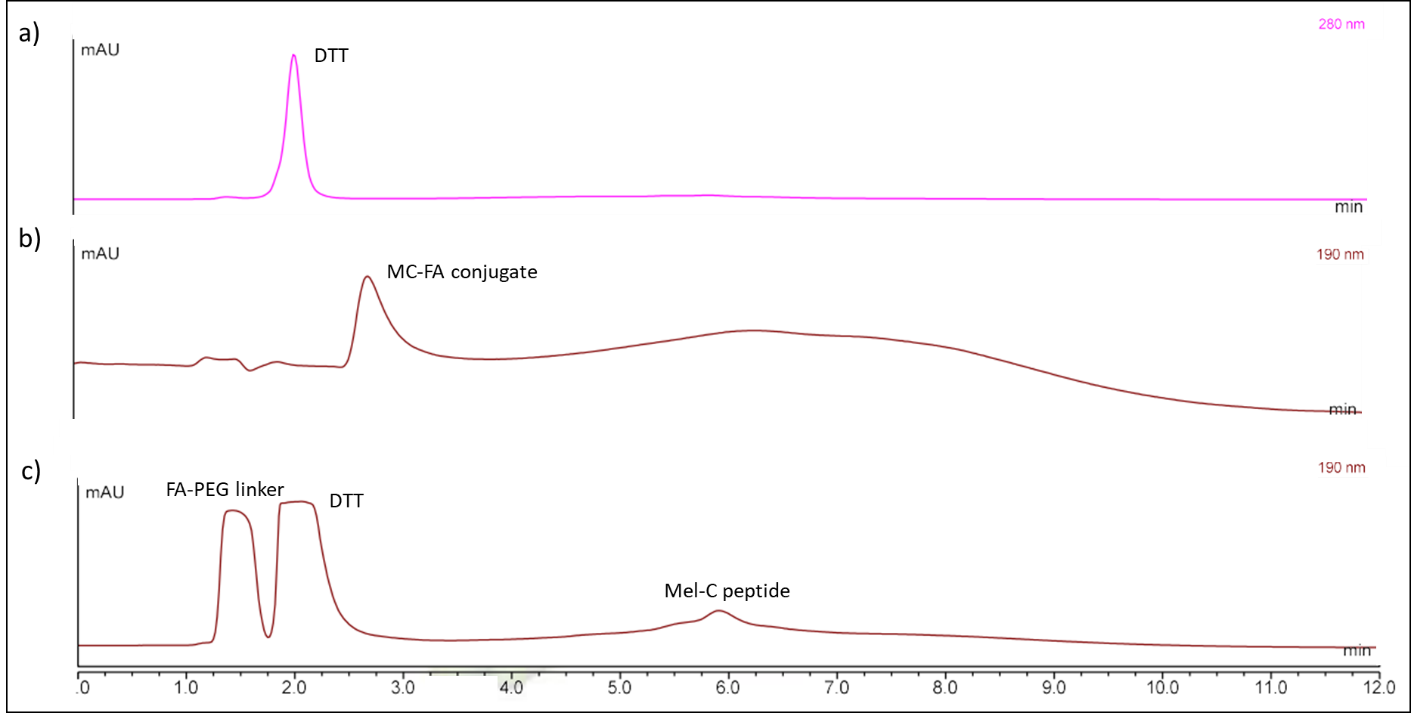


**Figure 3.** HPLC chromatogram of the redox-responsive linker cleavage. a) DTT alone, b) MC–FA conjugate with PBS, c) MC–FA conjugate with DTT.
